# Hypoxic postconditioning drives protective microglial responses and ameliorates white matter injury after ischemic stroke

**DOI:** 10.1111/cns.14346

**Published:** 2023-07-12

**Authors:** Wei Zhang, Sijie Li, Ho Jun Yun, Wantong Yu, Wenjie Shi, Chen Gao, Jun Xu, Yu Yang, Linhui Qin, Yuchuan Ding, Kunlin Jin, Fengyong Liu, Xunming Ji, Changhong Ren

**Affiliations:** ^1^ Beijing Key Laboratory of Hypoxia Translational Medicine, Xuanwu Hospital Capital Medical University Beijing China; ^2^ Department of Neurosurgery Wayne State University School of Medicine Detroit Michigan USA; ^3^ Department of Neurology, Xuanwu Hospital Capital Medical University Beijing China; ^4^ Center of Stroke, Beijing Institute for Brain Disorder Capital Medical University Beijing China; ^5^ School of Chinese Medicine Beijing University of Chinese Medicine Beijing China; ^6^ Department of Pharmacology and Neuroscience University of North Texas Health Science Center Fort Worth Texas USA; ^7^ Department of Interventional Radiology, Senior Department of Oncology Fifth Medical Center of PLA General Hospital Beijing China

**Keywords:** hypoxic postconditioning, ischemic stroke, MCAO, microglial, oligodendrocytes, white matter

## Abstract

**Background:**

Ischemic stroke (IS) is a cerebrovascular disease with high incidence and mortality. White matter repair plays an important role in the long‐term recovery of neurological function after cerebral ischemia. Neuroprotective microglial responses can promote white matter repair and protect ischemic brain tissue.

**Aims:**

The aim of this study was to investigate whether hypoxic postconditioning (HPC) can promote white matter repair after IS, and the role and mechanism of microglial polarization in white matter repair after HPC treatment.

**Materials & Methods:**

Adult male C57/BL6 mice were randomly divided into three groups: Sham group (Sham), MCAO group (MCAO), and hypoxic postconditioning group (HPC). HPC group were subjected to 45 min of transient middle cerebral artery occlusion (MCAO) immediately followed by 40 min of HPC.

**Results:**

The results showed that HPC reduced the proinflammatory level of immune cells. Furthermore, HPC promoted the transformation of microglia to anti‐inflammatory phenotype on the third day after the procedure. HPC promoted the proliferation of oligodendrocyte progenitors and increased the expression of myelination‐related proteins on the 14th day. On the 28th day, HPC increased the expression of mature oligodendrocytes, which enhanced myelination. At the same time, the motor neurological function of mice was restored.

**Discussion:**

During the acute phase of cerebral ischemia, the function of proinflammatory immune cells was enhanced, long‐term white matter damage was aggravated, and motor sensory function was decreased.

**Conclusion:**

HPC promotes protective microglial responses and white matter repair after MCAO, which may be related to the proliferation and differentiation of oligodendrocytes.

## Graphical abstract

Schematic diagram of experimental time. In the acute stage of cerebral ischemia, the function of proinflammatory immune cells is enhanced, the long‐term white matter injury is aggravated, and the motor sensory function is decreased. HPC reduces proinflammatory response and promotes protective microglia response and white matter repair after cerebral ischemia.

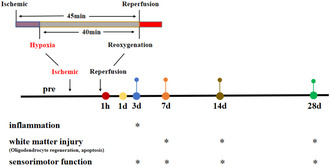

## 1 INTRODUCTION

Stroke is a cerebrovascular event leading to high mortality and disability worldwide and 87% of the cases is associated with cerebral ischemia.^1^ Currently, the internationally accepted treatment methods are thrombolysis and endovascular therapy. Due to the limited treatment time window and the risk of bleeding, only about 5% of the patients are effectively treated.^2^, ^3^ Despite decades of searching for new treatments, no significant progress has been made.^4^

White matter injury is an important pathological process after stroke and an important risk factor for poor long‐term prognosis.^5^, ^6^ Ischemic stroke (IS) severely affects the white matter integrity of the brain and exacerbates cognitive impairment.^6^, ^7^, ^8^ Studies have shown that treatments for white matter damage, such as oligodendrocyte regeneration, myelin regeneration, and axonal repair, can promote the recovery of IS.^9^, ^10^, ^11^, ^12^

The immune system plays important roles in alleviating brain damage and promoting tissue repair after IS attack.^13^, ^14^ For instance, activation and polarization of microglia, immune cells in the central nervous system (CNS), are critical for disease damage and repair during the development of IS.^15^ Studies have noted microglia as an important factor in the occurrence of numerous CNS diseases and a key target for treatment.^16^ There is cumulating evidence suggesting that microglial activation and polarization play an important role in white matter repair after stroke.^9^ Related experimental studies have shown that microglia with anti‐inflammatory phenotype promoted white matter repair and neuroprotection by reducing axonal damage and promoting myelin regeneration after stroke.^17^, ^18^, ^19^

Hypoxic postconditioning (HPC) is a sub‐fatal hypoxic exposure after severe ischemic/hypoxic injury. HPC is an important means for the body to generate endogenous defense mechanisms.^20^ Studies have shown that HPC can reduce cerebral tissue damage after ischemia.^21^ It has been suggested that HPC plays a neuroprotective role by increasing glycolysis and regulating neural stem cell metabolism.^22^ Relevant evidence has demonstrated the neuroprotective effect of HPC though the specific mechanism is not clear.

This study utilized HPC as the treatment for mice with middle cerebral artery transient ischemic injury to evaluate the level of inflammatory cells represented by microglia. The relationship and mechanism between microglia polarization and white matter lesions were analyzed. It was hypothesized that HPC plays a role in white matter damage repair by driving the protective microglial response. These findings may provide insights into the treatment of neuroinflammatory and white matter‐related neurological diseases.

## 2 MATERIALS AND METHODS

### 2.1 Animal

Male C57BL/6 mice (8–10 weeks, weight 20–22 g) were purchased from Beijing Vital River Laboratory Animal Technology Co. Ltd. Mice were housed in a 12‐h light–dark cycle with controlled cage temperature (23 ± 2°C) and humidity (40%–60%). The animal experimental design and protocol in this study were approved by the Comments of Animal Experiments and Experimental Animal Welfare Committee of Capital Medical University. All experimental procedures were performed in accordance with the “Administrative Regulations on Laboratory Animals” approved by the State Council of the People's Republic of China.

### 2.2 Middle cerebral artery occlusion model establishment

Transient focal ischemia was induced by right middle cerebral artery occlusion using the method utilized in a past study.^22^, ^23^ Briefly, a heparinized suture traveled from the external carotid artery through the internal carotid artery to the anterior cerebral artery and blocked the blood supply to the middle cerebral artery. During the procedure, the rectal temperature was maintained at 37 ± 0.5°C by a temperature‐controlled heating pad. The thrombus was removed and re‐perfused 45 min after Middle cerebral artery occlusion (MCAO). Mice were randomly divided into three groups: Sham group (Sham), MCAO group (MCAO), and hypoxic postconditioning group (HPC). All mice were injected intraperitoneally with 5‐bromo‐2‐deoxyuridine (BrdU, 50 mg/kg) twice daily from the 7th to the 14th day after the procedure.

### 2.3 Hypoxic postconditioning

Mice were treated with HPC immediately after ischemia and subsequently placed in a hypoxia chamber (Beijing Sub‐low Temperature Constant Oxygen Measurement Technology Development Center, CW‐2000). The oxygen level in the hypoxic chamber was set to gradually drop to 5% within 40 min. The total duration of the HPC was 40 min.

### 2.4 Rotarod test

The rotarod test was performed in the traditional fashion.^24^ Mice were placed on a rotarod, the rotational speed was accelerated from 0 revolutions per minute (rpm) to 30 revolutions per minute (rpm) for 1 min, and the speed was for the next 4 min. Mice were trained before MCAO until they could stay on the rotarod for 5 min. Postoperative tests were performed on days 1, 3, 7, 14, 21, and 28 after MCAO, and the latency to fall from the rotarod was recorded. Three trials were performed daily and the average incubation time was used for analysis.

### 2.5 Balance beam

The balance beam test detected the motor integration ability and coordination of mice. Mice were scored as follows: maintaining a stable balance (0 point); grasping one end of the beam (1 point); clinging to the beam with one limb slipping (2 points); holding the beam and rotating around the wood on both sides or fall for 60 seconds or more (3 points); trying to balance but sliding between 40 and 60 s (4 points); trying to balance but sliding between 20 and 40 s (5 points); being unable to balance and slide within 20 s (6 points).

### 2.6 ELISA

The experimental sample was mouse right brain tissue protein homogenate. The levels of inflammatory cytokines (IL‐1β, IL‐10, TGF‐β1, iNOS) were measured by ELISA detection kit (NeoBioscience Technology, China) using the double antibody sandwich method. Experimental procedures of the manufacturer's manual were strictly followed. Values were expressed in pictograms of cytokines per milligram (pg/mg).

### 2.7 Quantitative inflammation array

The tested brain tissues were harvested and the right infarcted brain tissue was homogenized. Once the array chip was completely dry, 100 μl of the tissue sample was added to the diluent in each well. Quantibody mouse inflammation with 40 Cytokines Array Kit was used according to the manufacturer's instructions (Ray Biotech, Inc.). Finally, the relative expression of each cytokine was calculated compared to the Sham‐operated group.

### 2.8 Luxol fast blue staining

Luxol fast blue (LFB) staining was used to demonstrate myelination. Brain tissue slices were incubated with LFB (Solarbio) solution at room temperature for 12–20 h. The staining solution was washed off with 95% alcohol and treated with Luxol differentiation solution for 15 s. Then, color separation was performed with 70% ethanol for 30 s. The positive deep staining area was the myelin sheath of brain tissue. Myelinated area (%) = Optical density of dark‐stained areas of the corpus callosum/Optical density of corpus callosal area × 100%.

### 2.9 Immunofluorescence staining

Brain sections were fixed in methanol and membranes were ruptured with 0.25% Triton‐X100. After blocking with 1% BSA, sections were incubated overnight at 4°C using the following primary antibodies: rabbit anti‐Iba‐1 (Wako), rat anti‐CD16 (BD biosciences), goat anti‐CD206 (R&D Systems), rat anti‐F4/80 (BioLegend), rabbit anti‐MPO (Abcam), rabbit anti‐MBP (Abcam), mouse anti‐NF200 (SigmaAldrich), mouse anti‐BrdU (GeneTex), and rabbit anti‐CNPase (Abcam). TUNEL assays were performed using in situ cell death detection kit, fluorescein (Roche). Positive cells from the peripheral areas of the infarct core were obtained from the microscope field, including the corpus callosum and striatum in the white matter area. Nine images were randomly selected for each region for calculation.

### 2.10 Western blot

Right mouse brain tissue was homogenized in RIPA lysis buffer containing protease inhibitors. The supernatant was collected after centrifugation and the protein concentration of the samples was determined by BCA protein assay kit (Bio‐Rad). Proteins were separated by polyacrylamide gel electrophoresis and transferred to cellulose acetate membranes. After blocking with 5% milk for 1 h, the primary antibody was incubated overnight. The primary antibodies were rabbit anti‐MBP (Abcam), rabbit anti‐β‐actin (Santa Cruz), rabbit anti‐PDGFR‐α (Cell Signaling), and rabbit anti‐APC (SigmaAldrich). Horseradish peroxidase‐conjugated secondary antibody (HRP, Abgent) was incubated for 1 h at room temperature. Protein bands were exposed using chemiluminescent reagents (Millipore). Image capture was performed with a chemiluminescent gel imager. Protein gray values were calculated with image j analysis software and corrected with β‐actin. The grayscale of the protein was expressed in relative values.

### 2.11 Statistical analysis

Data were expressed as means ± standard deviations. Each graph provides a sample size. Shapiro–Wilk tests were used to determine the normality of the data. For normally distributed data, Student's *t* test (2 groups) or one‐way analysis of variance was used, followed by Tukey *post hoc* test (>2 groups). For the data that failed the normality test, the Mann–Whitney test (2 groups), or the Kruskal–Wallis test, and the Dunn post‐test (>2 groups) were used. Behavioral test data were analyzed by bidirectional analysis of variance and Bonferroni post hoc test. The data were analyzed using SPSS 22.0. *p* ≤ 0.05 was considered statistically significant.

## 3 RESULTS

### 3.1 HPC promotes early and long‐term recovery of motor sensory function after MCAO

**FIGURE 1:**
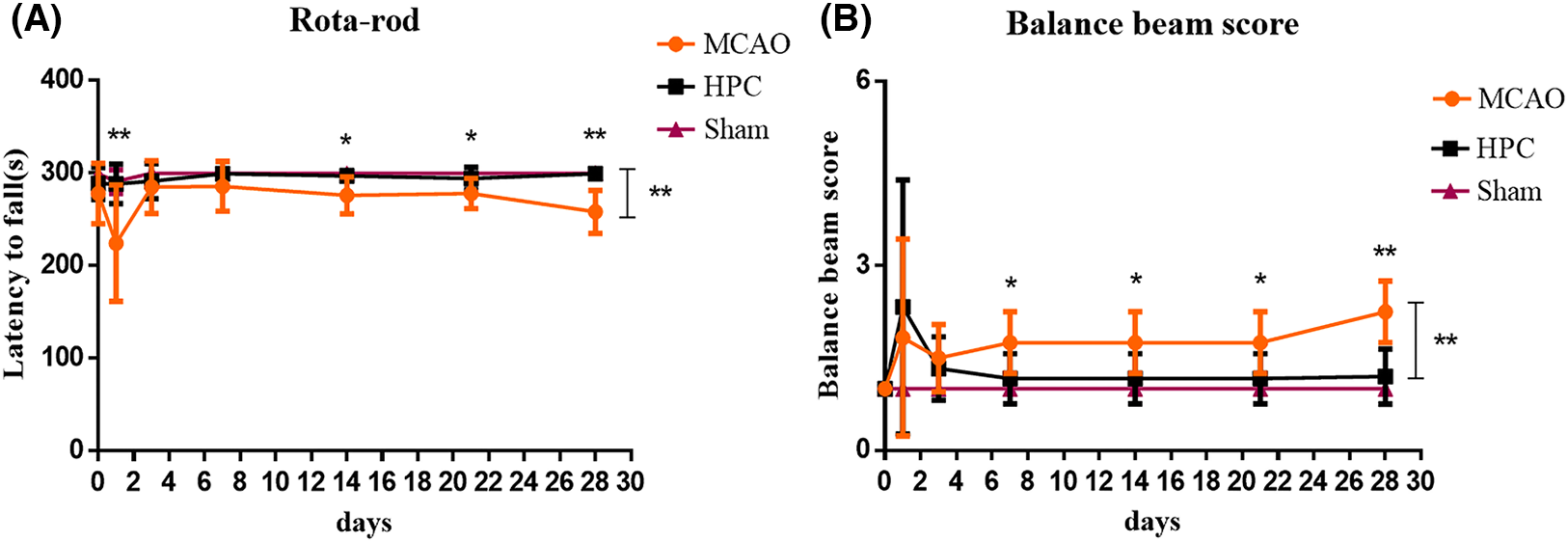
HPC significantly promotes long‐term motor function recovery after focal cerebral ischemia. (A, B) Neurobehavioral experiments were performed on days 1, 3, 7, 14, 21, and 28 after MCAO. (A) Rotarod test. (B) Balance beam test. Bar graphs are shown with mean ± SD. *n* = 5 mice per group. *p ≤ 0.05; **p ≤ 0.01; ***p ≤ 0.001.

In order to verify the effect of HPC on motor sensory recovery after MCAO, cerebral infarction was induced to mice by MCAO and they were treated with HPC for 40 min immediately. The neuroprotective effect of HPC was evaluated by the long‐term recovery of motor sensory function. Our previous study showed that HPC improved neurological function in MCAO mice. Therefore, rotarod test and balance beam were used to examine the recovery of motor sensory function of mice. Compared with the MCAO model group, HPC increased the latency to drop in the rotarod test (Figure 1A) and the stability of the balance beam (Figure 1B). These experimental results suggested that HPC provides early and long‐term neuroprotective effects after MCAO.

### 3.2 HPC reduces immune cell infiltration after MCAO and inhibits proinflammatory responses in the brain

**FIGURE 2:**
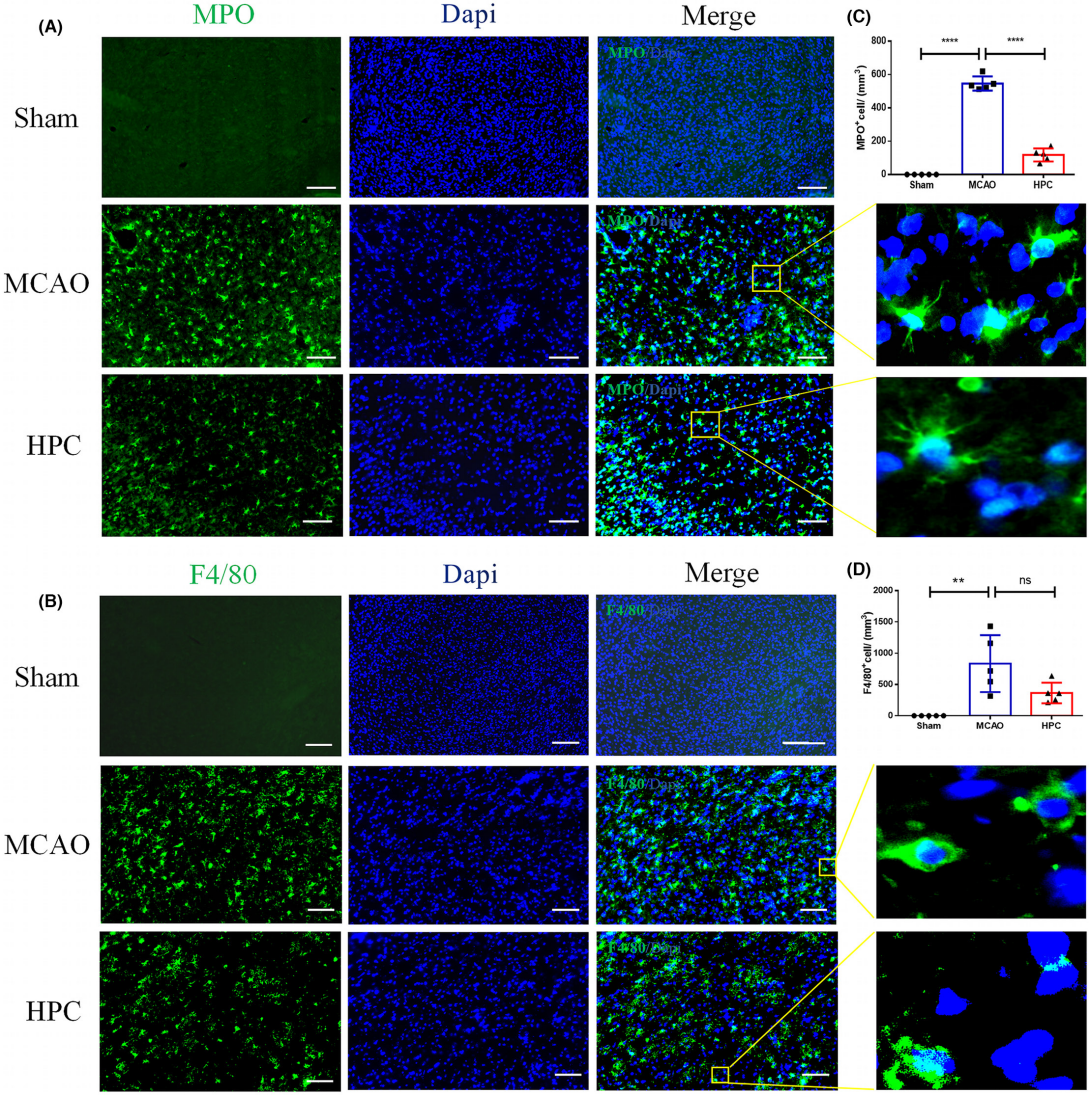
HPC reduces immune cell infiltration in the brain after MCAO. Mice followed 3 days after MCAO (I/R). (A, B) Images of cortical and striatum positive cells from the periphery of the infarct core staining for the following markers: MPO (green, neutrophil), F4/80 (green, macrophage), and Dapi (blue, nucleus). Scale bar, 100 mm. (A) Images of MPO (green, neutrophil) and Dapi (blue, nucleus). Square: the region enlarged in high‐power images. (B) Images of F4/80 (green, macrophage), Dapi (blue, nucleus). Square: the region enlarged in high‐power images. (C) MPO^+^ cells were counted in the described areas and data were expressed in number of cells per mm^3^. (D) F4/80^+^ cells were counted in the described areas and data were expressed in number of cells per mm^3^. Bar graphs are shown in mean ± SD. *n* = 5 mice per group. ns: no significance, *p ≤ 0.05; **p ≤ 0.01; ***p ≤ 0.001.

The immune system plays an important role in the pathological process of IS. Brain cells activate the immune system upon injurious stress, leading to the infiltration of immune cells and triggering a proinflammatory response.^25^ The experimental results have shown that, in the early stage of ischemia, the infiltration of neutrophils and monocytes occurs. HPC treatment could reduce the expression of neutrophils (Figure 2A,C).

### 3.3 HPC promotes the transformation of microglia to anti‐inflammatory phenotype after ischemia–reperfusion

**FIGURE 3:**
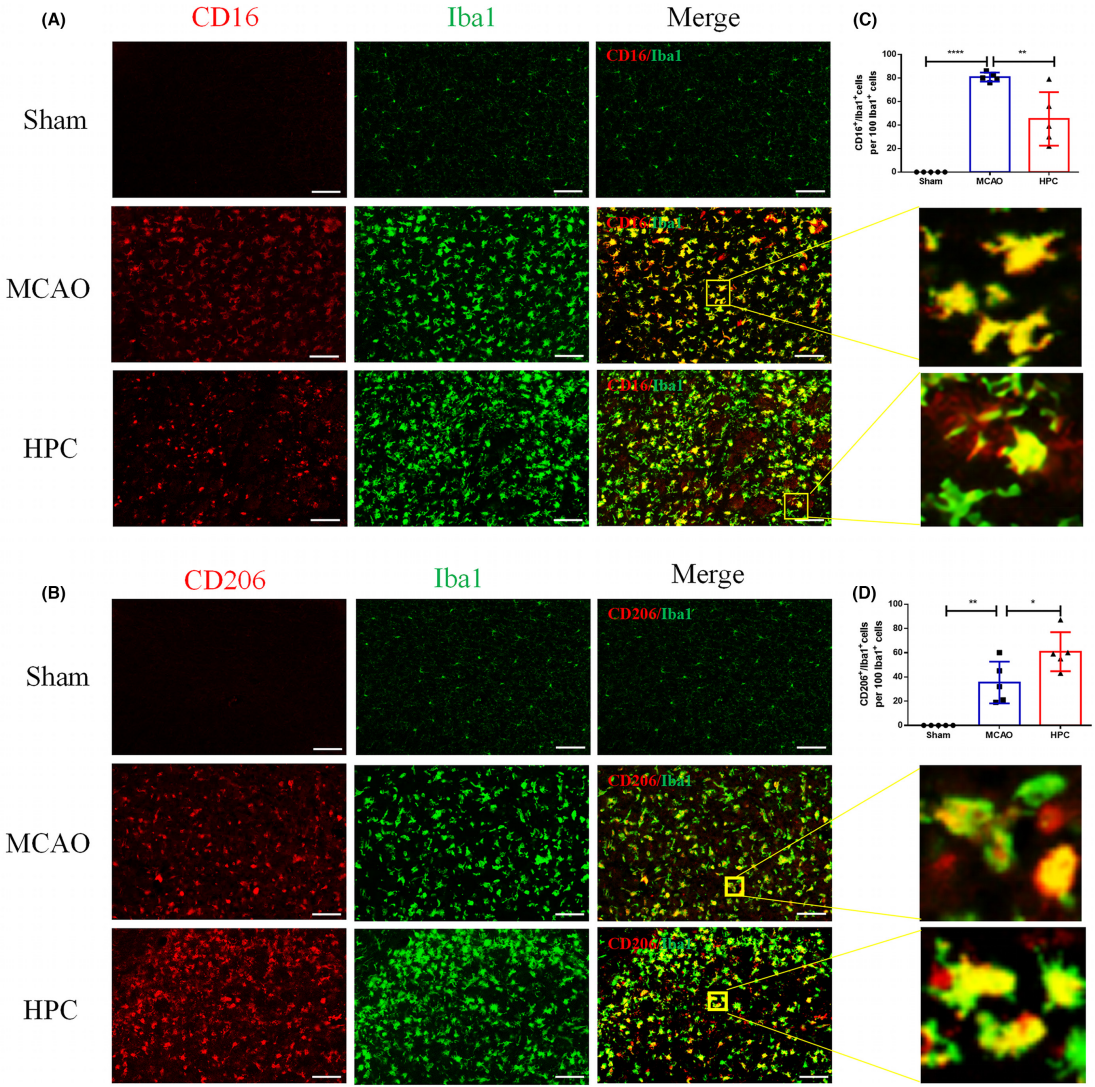
HPC promotes a shift toward anti‐inflammatory phenotypes in microglia/macrophages in the sub‐acute stage after MCAO. Mice followed 3 days after MCAO (I/R). (A, B) Images of cortical and striatum positive cells from the periphery of the infarct core for the following markers: Iba1 (green, Microglial), CD16 (red, proinflammatory phenotypic marker), CD206 (red, anti‐inflammatory phenotypic marker). Scale bar, 100 mm. (A) Images of Iba1 (green, Microglial) and CD16 (red, proinflammatory phenotypic marker). Square: the region enlarged in high‐power images show cell morphology. (B) Images of Iba1 (green, Microglial) and CD206 (red, anti‐inflammatory phenotypic marker). Square: the region enlarged in high‐power images. (C) Iba1^+^ cells and CD16^+^/Iba1^+^cells were counted in the described areas and data were expressed in number of CD16^+^/Iba1^+^cells per 100 Iba1^+^ cells. (D) Iba1^+^ cells and CD206^+^/Iba1^+^cells were counted in described areas and data were expressed in number of CD206^+^/Iba1^+^cells per 100 Iba1^+^ cells. Bar graphs are shown in mean ± SD. *n* = 5 mice per group. *p ≤ 0.05; **p ≤ 0.01; ***p ≤ 0.001.

**FIGURE 4:**
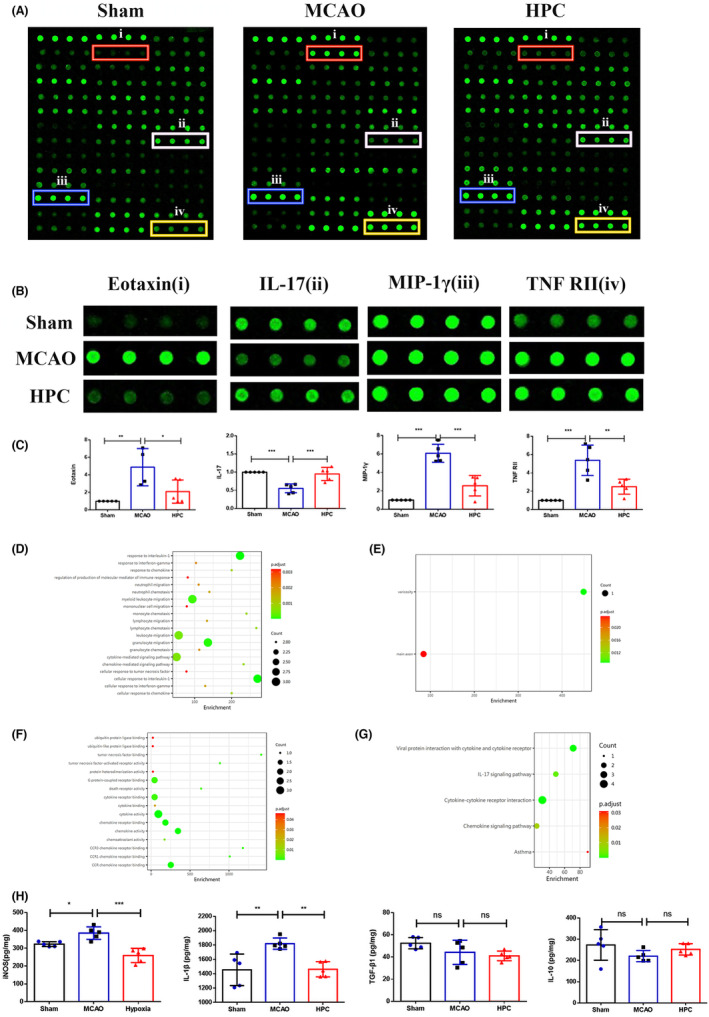
HPC regulates the expression of inflammatory factors. Mice followed 3 days after MCAO (I/R). A group of inflammatory markers was examined using the quantitative inflammation array in the ischemic hemisphere on day 3 after IS. (A) Representative images of the inflammation array in the Sham, MCAO, and HPC group. (B) Images of differently expressed inflammatory factors: eotaxin, MIP‐1γ, TNF RII, and IL‐17. (C) Data were expressed in levels of inflammatory markers relative to Sham. (D–G) GO and KEGG analysis of the quantitative inflammation array. GO analysis included cellular component, molecular function, and biological process analysis. (D) biological process. (E) cellular component. (F) molecular function. (G) KEGG analysis. (H) Quantification of ELISA for inflammatory cytokines (IL‐1β, IL‐10, TGF‐β1, and iNOS). Bar graphs are shown in mean ± SD. *n* = 5 mice per group. ns: no significance, *p ≤ 0.05; **p ≤ 0.01; ***p ≤ 0.001.

Microglia are the main immune cells involved in the defense mechanism against brain damage.^26^ MCAO ischemia–reperfusion (I/R) stimulation produces M1 phenotype expressing proinflammatory cytokines and produces M2 phenotype expressing repair factors.^27^ In fact, conversion from type M1 of microglia to M2 indicates nerve repairmen. To investigate the neural repairing effect of HPC, this study used immunofluorescence double‐labeled Iba1/CD16 and Iba1/CD206 microglia representing different phenotypes. Mice followed 3 days after MCAO (I/R). Compared with the MCAO group, the M1‐phenotype microglia decreased after HPC treatment (Figure 3A). In HPC group, M2 microglia expressing repair factors were significantly increased (Figure 3B). Additionally, this experiment detected cytokines (IL‐1β, IL‐10, TGF‐β, iNOS) and 40 other inflammatory markers. Relative quantification by protein array showed that HPC decreased the expression of eotaxin, MIP‐1γ, and TNF RII, and increased the expression of IL‐17 (Figure 4A–C). GO and KEGG analysis showed that the therapeutic effect of HPC was related to viral protein interaction with cytokine and cytokine receptor and IL‐17 signaling pathway (Figure 4D–G). Furthermore, the expression of iNOS and IL‐1β in HPC group was lower than the MCAO group (Figure 4H). The results confirmed that HPC played a neuroprotective role by promoting the transformation of microglia into anti‐inflammatory phenotype after MCAO (I/R).

### 3.4 HPC promotes white matter repair 14 days after I/R

**FIGURE 5:**
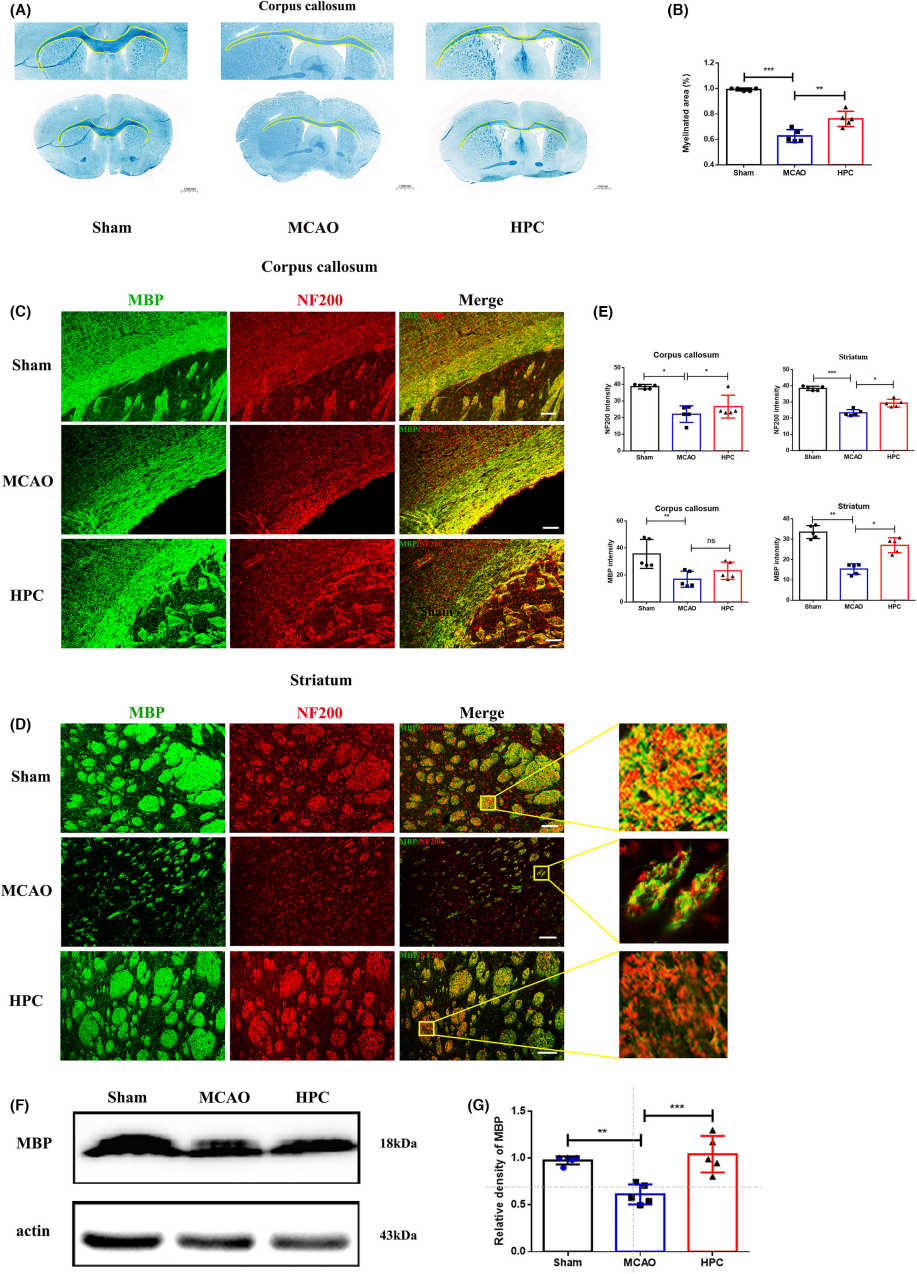
HPC attenuates white matter lesions on the 14th day after IS. Mice followed 14 days after MCAO (I/R). Positive cell images from the peripheral areas of the infarct core were obtained, including the corpus callosum and striatum in the white matter area. (A) White matter lesions were detected by LFB staining in different groups. The area circled by the white line is the white matter defect and the area circled by the yellow line is the white matter. (B) Quantification of the OD value of LFB in corpus callosum. The integrated density of LFB staining was quantified and normalized to the Sham group. (C, D) Images of MBP (green, myelin basic protein) and NF200 (red, nerve fiber protein) in the corpus callosum and striatum. Scale bar, 100 mm. Square: The enlarged regions in the high magnification images demonstrate morphological features. (E) MBP and NF200 immunofluorescence density was calculated as a parameter for white matter integrity. (F) Representative WB bands of MBP. (G) Quantitative analyses of MBP expression. Bar graphs are shown in mean ± SD. *n* = 5 mice per group. ns: no significance, *p ≤ 0.05; **p ≤ 0.01; ***p ≤ 0.001.

HPC can play a role in repairing the injured brain through microglial polarization in the acute phase after MCAO (I/R). Relevant studies have shown that the transformation of microglia into anti‐inflammatory phenotype contributes to the long‐term repair of white matter injury.^28^ In order to test whether HPC can be transformed into anti‐inflammatory phenotype by microglia and play a role in long‐term repair of white matter injury in brain tissue, white matter integrity was observed with LFB staining 14 days after MCAO (I/R) and detection of MBP, NF200, and other proteins. Compared with the MCAO group, the optical density of LFB stain in the corpus callosum of mice treated with HPC was significantly increased (Figure 5A,B). The expression levels of MBP and NF200 in the striatum were also significantly increased in the HPC group (Figure 5D,E). Protein quantification results showed that the expression of MBP in the HPC group was higher than that in the MCAO group (Figure 5F,G). These results indicated that HPC treatment significantly increased the expression levels of proteins related to myelin integrity, and contributed to myelin repair after IS.

### 3.5 HPC promotes white matter repair 28 days after I/R

**FIGURE 6:**
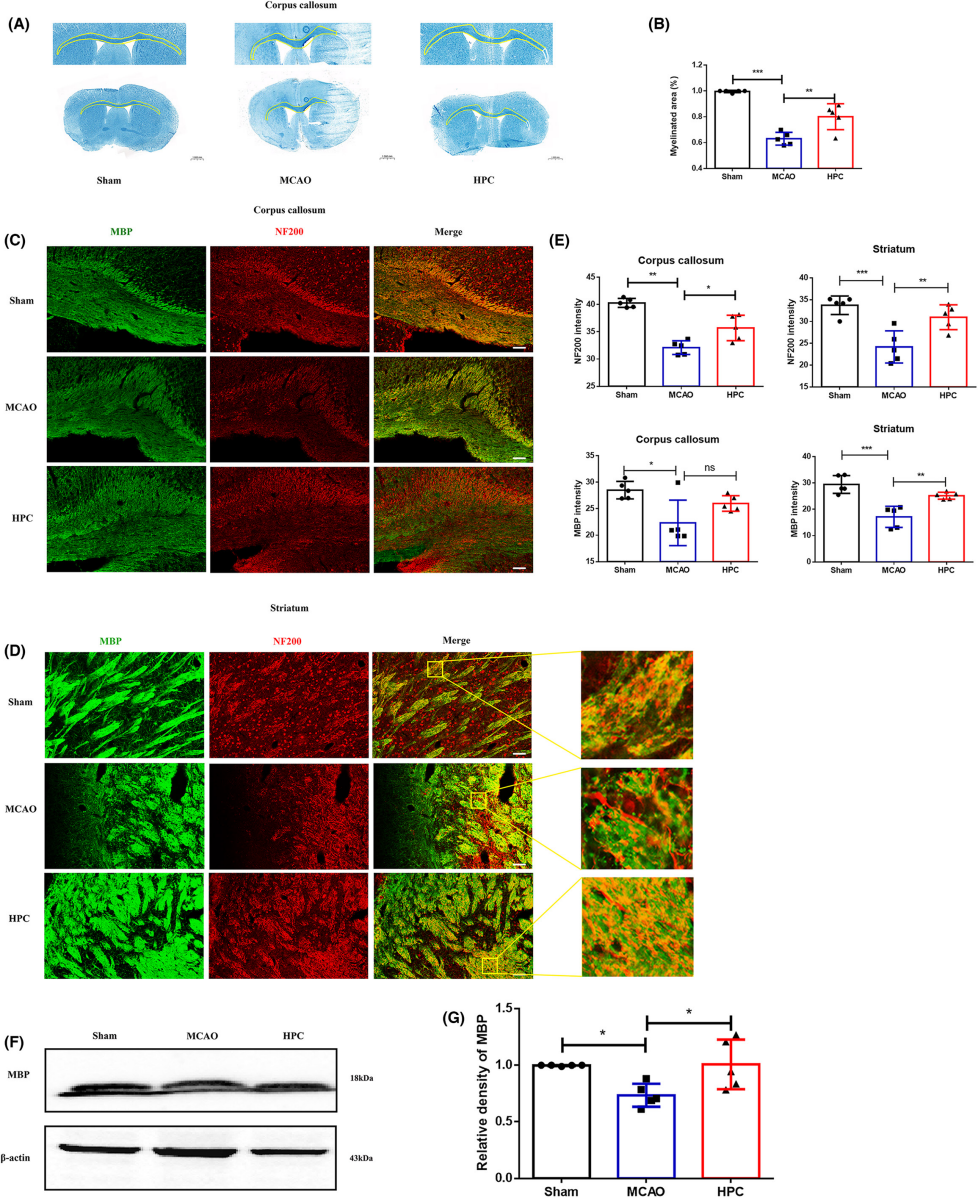
HPC attenuates white matter lesions in the chronic stages after IS. Mice followed 28 days after MCAO (I/R). Positive cell images from the peripheral areas of the infarct core were obtained, including the corpus callosum and striatum in the white matter area. (A) White matter lesions were detected by LFB staining in different groups. The area circled by the white line is the white matter defect, and the area circled by the yellow line is the white matter. (B) Quantification of the OD value of LFB in corpus callosum. The integrated density of LFB staining was quantified and normalized to the Sham group. (C, D) Representative images of MBP (green, myelin basic protein) and NF200 (red, nerve fiber) in the corpus callosum and striatum. Scale bar, 100 mm. Square: The enlarged regions in the high magnification images demonstrate morphological features. (E) MBP and NF200 immunofluorescence density was calculated as a parameter for white matter integrity. (F) Representative WB bands of MBP. (G) Quantitative analyses of MBP expression. Bar graphs are mean ± SD. *n* = 5 mice per group. ns: no significance, *p ≤ 0.05; **p ≤ 0.01; ***p ≤ 0.001.

The above results confirmed that HPC has a white matter repair effect 14 days after MCAO (I/R). In order to investigate whether HPC has sustained repair effect on the brain tissue, the study performed firm blue staining and measured MBP and NF200 proteins from the brain tissue 28 days after MCAO (I/R). Compared to the MCAO group, HPC could not only enhance the optical density of LFB staining (Figure 6A,B) but also increase the expression of MBP and NF200 (Figure 6C–G). These findings demonstrated that HPC had a protective effect on brain tissue in the acute phase of I/R as well as a long‐term repair effect on white matter damage.

### 3.6 HPC has no significant effect on axonal injury and oligodendrocyte apoptosis 7 days after I/R

**FIGURE 7:**
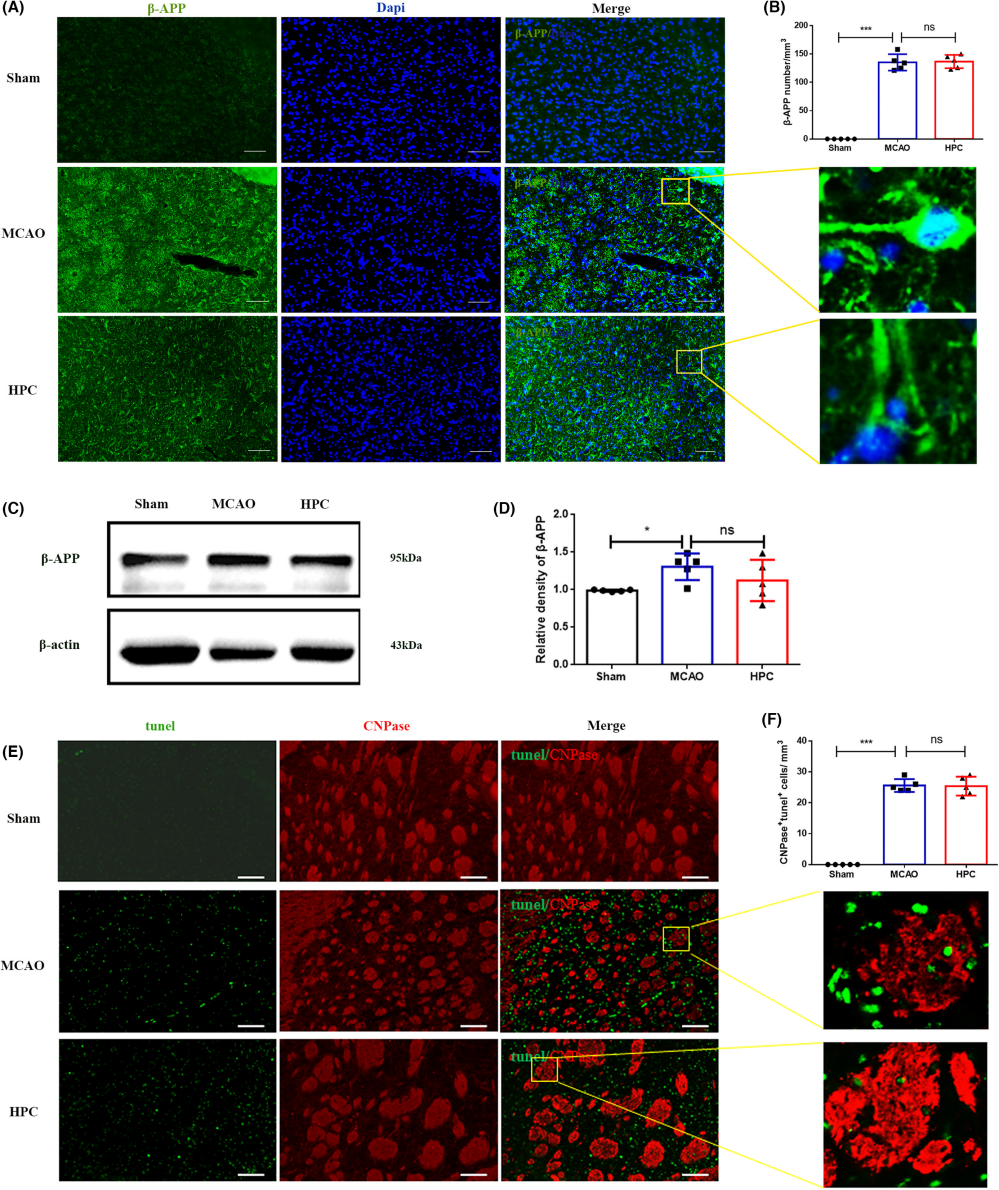
HPC has no significant effect on axonal injury and oligodendrocyte apoptosis on the 7th day after I/R. Mice followed 7 days after MCAO (I/R). (A) Positive cell images were obtained from the peripheral striatum area of the infarct core. Showing immunofluorescence staining for the following markers: β‐APP (green, axonal injury marker), Dapi (blue, nucleus). Square: the region enlarged in high‐power images. (B) Data were expressed in number of β‐APP^+^ cells per mm^3^. (C) Representative WB bands of β‐APP. (D) Quantitative analyses of β‐APP expression. (E) Images from the inner border of infarction in the ipsilateral peri‐infarct regions. Showing immunofluorescence staining for the following markers: tunel (green, apoptosis marker), CNPase (red, oligodendrocyte). (F) Data were expressed in number of tunel ^+^/CNPase^+^ cells per mm^3^. Scale bar, 100 mm. Bar graphs are shown in mean ± SD. *n* = 5 mice per group. ns: no significance, *p ≤ 0.05; **p ≤ 0.01; ***p ≤ 0.001.

The above findings demonstrated that HPC could induce the anti‐inflammatory phenotype of microglia and play a role in long‐term repair of the white matter of damaged brain tissue. To explore the specific biological process of the effect, it was questioned whether HPC could also inhibit apoptosis and axonal injury during the acute phase after MCAO. The expression of β‐APP (a marker of axonal injury), was also not significantly decreased after HPC, compared to the MCAO group and the difference was not statistically significant (Figure 7A–D). Tunel^+^/CNPase^+^(an oligodendrocyte marker of apoptosis) was not significantly reduced after HPC treatment compared to the MCAO group (Figure 7E,F). The findings demonstrated that HPC had no significant effect on inhibiting apoptosis and axonal injury in the acute phase after MCAO injury.

### 3.7 HPC promotes long‐term white matter repair after I/R by promoting oligodendrocyte differentiation and maturation

**FIGURE 8:**
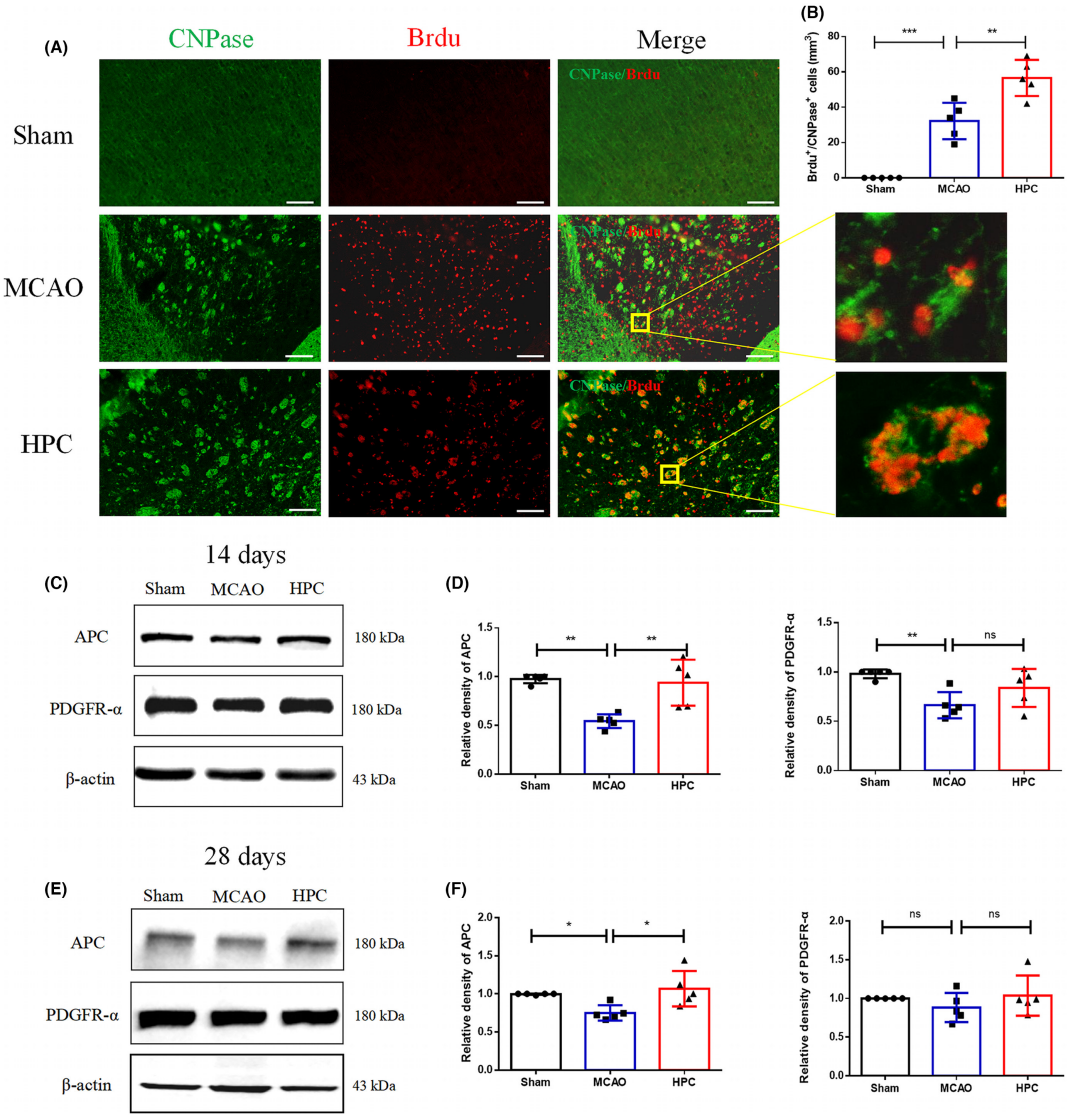
HPC promotes oligodendrocyte differentiation and maturation. (A) Mice followed 14 days after MCAO (I/R). Positive cell images were obtained from the peripheral striatum area of the infarct core. Showing immunostaining for the following markers: CNPase (green, oligodendrocyte), Brdu (red, neogenesis marker). Scale bar, 100 mm. (B) Data were expressed in number of Brdu^+^/CNPase^+^ cells per mm^3^. (C) Representative WB bands of APC and PDGFR‐α on the 14th day after IS. (D) Quantitative analyses of APC and PDGFR‐α expression. (E) Representative WB bands of APC and PDGFR‐α on the 28th day after IS. (F) Quantitative analyses of APC and PDGFR‐α expression. Bar graphs are shown in mean ± SD. *n* = 5 mice per group. ns: no significance, *p ≤ 0.05; **p ≤ 0.01; ***p ≤ 0.001.

It was hypothesized that HPC could play a role in long‐term white matter repair after I/R by promoting the differentiation and maturation of oligodendrocytes. The levels of PDGFR‐α (oligodendrocyte precursor cell marker) and APC (mature oligodendrocyte marker) were measured on days 14 and 28 after MCAO (I/R). Elevation of these proteins level could represent the differentiation and maturation of microglia. The expression of APC after MCAO was increased on day 14 after MCAO (I/R) (Figure 8C,D). Their expression also significantly increased on day 28 after MCAO (I/R) (Figure 8E,F). This finding confirmed that HPC can transform microglia into anti‐inflammatory phenotype. HPC played a role in long‐term repair of white matter injury in brain tissue by promoting oligodendrocyte differentiation and maturation.

## 4 DISCUSSION

This study demonstrated that HPC starting from 5 min after the onset of MCAO dramatically improved an array of histopathological and behavioral outcomes at acute and subacute injury stages. Collectively, the data presented here reveal the following findings. First, HPC can reduce the proinflammation cell level in the acute phase of cerebral ischemia. At the same time, HPC promotes the polarization of microglia toward the anti‐inflammatory phenotype. Second, HPC increases myelin integrity and has a long‐term repair effect on white matter damage in ischemic brain tissue. Additionally, the differentiation and maturation of oligodendrocytes play an important role in the repair of white matter damage in the chronic phase of IS. These results suggest that HPC has vital roles in long‐term repair of white matter injury and drives microglial neuroprotective responses in the acute phase of IS.

HPC appears to have specific protective effects on tissue injury after cerebral ischemia. A study has recently shown that HPC has a neuroprotective role after cerebral ischemia by inducing mitophagy in the hippocampus.^29^ Related experiments have confirmed that HPC reduces neuronal death after transient ischemic attack (TIA).^30^ Additionally, HPC has impacts on vessels and has been found to prevent vascular embolism and spasm after ischemia.^31^ In this study, HPC was found to reduce the proinflammatory response during the acute phase of cerebral ischemia. Elevations of eotaxin, MIP‐1γ, and TNF RII, or proinflammatory cytokines were associated with poor long‐term recovery.^32^, ^33^, ^34^ HPC reduced the expression of eotaxin, MIP‐1γ, and TNF RII after MCAO. Eotaxin belongs to the CC‐chemokine family and induces an eosinophil response.^35^ It plays an important role in the pathogenesis of IS due to its ability to cross the blood–brain barrier.^36^ The level of eotaxin increases with the occurrence of IS, and the increase in eotaxin aggravates IS injury.^37^ Macrophage inflammatory protein‐1 gamma (MIP‐1γ) also belongs to the CC‐chemokine family. MIP‐1γ promotes macrophage cell recruitment and aggravates the inflammation of brain tissue after IS.^38^ The MIP‐1γ level decreased when brain tissue damage was reduced.^39^ Cytokine tumor necrosis factor (TNF) is considered to be a cytokine highly associated with IS.^40^ TNFRII is a receptor of TNF. The level of TNFRII is associated with stroke severity and prognosis.^41^ Therefore, the decreased expression of eotaxin, MIP‐1γ, and TNF RII further confirmed the neuroprotective effect of HPC.

The results of this study showed that the neuroprotective effect of HPC was not only from reducing the proinflammatory response but also promoting microglial responses. KEGG enrichment analysis showed that the anti‐inflammatory effect of HPC was highly correlated with IL‐17A signaling pathway. IL‐17A was found to promote the polarization of macrophages to M2 type.^42^ Activation of microglia can attenuate neuronal apoptosis, enhance neurogenesis, and promote recovery from cerebral ischemia.^43^, ^44^, ^45^, ^46^

Microglia are central nervous system macrophages, which play an important role in maintaining local brain microenvironment homeostasis and are the main cells mediating the inflammatory response after stroke.^47^, ^48^ Related experiments have found that inhibition of microglial thermal sagging can also improve cerebral ischemic injury.^17^ This study has confirmed that HPC promotes the transformation of microglia into an anti‐inflammatory phenotype in the acute phase of cerebral ischemia and clarified HPC can play a long‐term protective role in brain injury by promoting microglia polarization.

The white matter of CNS is mainly composed of myelin sheaths and axons that are responsible for nerve electrical signal conduction.^49^ IS commonly causes white matter injury and white matter lesions are closely related to the prognosis of endovascular treatment after IS.^50^ Studies have shown that microglia can mediate the white matter repair after IS.^9^ This study demonstrated that increased expression of MBP and NF200 by HPC had a restorative effect on white matter damage 14 and 28 days after cerebral ischemia. HPC not only repaired the white matter structure but also greatly improved the motor behavior of mice after ischemia. Multiple factors affect the integrity of white matter after stroke: oligodendrocyte apoptosis, oligodendrocyte regeneration, differentiation, and maturation.^51^ The findings showed that HPC had no significant effect on oligodendrocyte apoptosis. However, HPC promoted oligodendrocyte regeneration, differentiation as well as maturation. The role of oligodendrocytes is to produce myelin, or an essential component of white matter.^52^ In the adult brain, there are mature oligodendrocytes as well as oligodendrocyte progenitors.^53^ Oligodendrocyte precursor cells can proliferate, differentiate, and become mature to promote myelination during the process of injury repair.^54^ The findings of this study confirmed that HPC can increase the regeneration, differentiation, and maturation of oligodendrocyte progenitors after cerebral ischemia, which promotes myelination and white matter repair.

Microglia have been found to promote myelination and repair by regulating the proliferation of oligodendrocyte precursor cells.^55^ Repot showed that increasing the activity of tissue‐repairing microglia can up‐regulate the expression of cytokines that promote oligodendrocyte production in microglia, thereby promoting oligodendrocyte regeneration and myelination in the chronic recovery period of stroke, and thus improving neurological function.^9^ In this study, HPC therapy has also been shown to decrease the number of CD16^+^ proinflammatory microglia/macrophages, which are associated with white matter injury. Therefore, we hypothesized that the promotion of white matter damage repair by HPC may be related to its role in regulating microglial polarization, which needs further investigation.

In conclusion, HPC plays a role in white matter repair after IS by inhibiting proinflammatory response and increasing microglial neuroprotective responses. The process of white matter repair may be related to the proliferation and differentiation of oligodendrocytes.

## AUTHOR CONTRIBUTIONS

All the authors had full access to all study data and take responsibility for the integrity of the data and the accuracy of the data analyses.

## FUNDING INFORMATION

This work was supported by the National Natural Science Foundation of China (grant numbers 81971114 and 82274401).

## CONFLICT OF INTEREST STATEMENT

The authors confirm that they have no conflict of interest.

## Data Availability

The data that support the findings of this study are available from the corresponding author upon reasonable request.
